# A Bionic Walking Wheel for Enhanced Trafficability in Paddy Fields with Muddy Soil

**DOI:** 10.3390/biomimetics9020068

**Published:** 2024-01-24

**Authors:** Duo Chen, Yan Xu, Yuqiu Song, Mingjin Xin, Liyan Wu, Aiju Kong, Huan Wang, Pengchao Dai, Hongpeng Yu

**Affiliations:** 1 College of Engineering, Shenyang Agricultural University, Shenyang 110866, China; chenduo@stu.syau.edu.cn (D.C.); songyuqiu@syau.edu.cn (Y.S.); xinmjsynd@163.com (M.X.); wly78528@syau.edu.cn (L.W.); kongaiju@syau.edu.cn (A.K.); syeng94277@syau.edu.cn (H.W.); dpc004@stu.syau.edu.cn (P.D.); 2 Shenyang Institute of Automation, Chinese Academy of Sciences, Shenyang 110016, China; yuhongpeng@sia.cn

**Keywords:** bionic walking wheel, paddy soils, tractability, waders, wheel–soil interaction

## Abstract

To improve wheel trafficability in soft and muddy soils such as paddy fields, a bionic walking wheel is designed based on the structural morphology and movement mode of the feet of waders living in marshes and mudflats, similar to the muddy soil of paddy fields. The bionic walking wheel adopts the arrangement of double-row wheel legs and staggered arrays to imitate the walking posture of waders. The two legs move alternately, cooperate with each other, and improve the smoothness of movement. The cam inside the bionic walking wheel is used to control the movement mode of the feet. The flippers open before touching the ground to increase the contact area and reduce sinking, and the toes bend and grip the ground while touching the ground to increase traction. Multi-rigid-body dynamics software (Adams View 2020) is used to simulate the movement of the wheel during the wading process, and the movement coordination and interference between the wheel legs are analyzed. The simulation results show that there is no interference between the parts and that the movement smoothness is good. The interaction between the bionic walking wheel and muddy soil was analyzed via coupled EDEM–ADAMS simulation, and the simulation analysis and experiments were conducted and compared with those for a common paddy wheel. The results showed that the bionic walking wheel designed in this paper improved the drawbar pull by 113.56% compared with that of a common paddy wheel and had better anti-sinking performance. By analyzing the effect of toe grip on traction, it was found that the soil under the feet can be disturbed to provide greater traction when the toe is bent downward. This study provides a reference for improving the trafficability of walking mechanisms in soft and muddy soils, such as paddy fields.

## 1. Introduction

Soft ground is generally characterized by high porosity and poor compactness of soil particles, resulting in low pressure-bearing capacity and shear strength [1]. The main source of traction force on the walking mechanism is the friction between the wheels and the soil and the shear force on the soil [2]. Paddy soil is different from common soft soil because of its solid–liquid mixed state and certain fluidity, plasticity, and viscosity. Moreover, this exacerbates the problem of insufficient shear resistance, often leading to sinking, slipping, or even the inability to travel [3].

Through natural selection, we found that some creatures can survive on muddy and soft ground. Wading birds live in swamps and wetlands throughout the year, and they can walk freely on muddy and soft ground, similar to paddy fields [4]. Owing to their unique foot structure and movement mode, these individuals can have better tractability on the ground. The analysis of the foot structure, movement mode, and interaction with the soil, according to the principle of bionics, can provide a certain theoretical basis for the bioinspired design of a walking mechanism on paddy soil using the movement mechanism of wading birds on soft and muddy ground [5].

The soft ground environment is roughly categorized into three main types: lunar surface, desert, and paddy field. To solve the problem of the traction of walking mechanisms in soft ground, scholars have performed considerable research. Zhao Yibing designed a half-step walking wheel based on the ground mechanics of the vehicle and derived the traction formula for the walking wheel. By simulating and analyzing the motion performance of a walking wheel, its smoothness and obstacle-crossing ability were verified [6]. Chen Dexing summarized the influence of traces on the ground surface after walking on the traction performance of different wheel structures and designed a transmission walking wheel with retractable legs and feet, which greatly improved the smoothness of the vehicle [7]. Chen Xinbo investigated the dynamic response of a wheel-body system during the process of changing the diameter of the wheel body to a walking wheel using rigid-flexible coupling simulation analysis, which provides a design reference for the transformed wheel-leg structure [8]. Lu Yang proposed a walking wheel for a lunar rover based on scalable blades, which was optimized by analyzing the mechanical properties of tires during movement. The blades can be stretched out as needed, reducing energy consumption and providing high traction and load-bearing performance [9]. Wei Cangang used the discrete element method (DEM) to simulate the traction performance of lunar rover wheels and studied the effect of different parameters on the performance of the wheels. Compared with the finite element method (FEM), the DEM is more suitable for simulating the behavior of discrete objects, such as lunar soil [10].

In addition, many scholars have begun to design walking wheels based on bionic principles. Hao Pang et al. designed a bionic tire based on the African ostrich as a prototype. The traction and torque of the bionic wheel and the wheel with a rectangular pattern at different slip rates are compared via DEM, and the results showed that the bionic wheel has better traction and anti-sinking properties in sand [11]. Zhang Rui designed a bionic walking wheel with different attitudes toward the wheel-leg structure using an ostrich as the bionic object. The effects of traction efficiency and sinking degree of wheel legs with different attitudes are tested through comparative experiments, and the results show that bionic wheel legs can effectively simulate the process of ostrich walking on sand and can improve the performance of vehicles on soft ground [12,13]. Inspired by the lateral fluctuating motion of snakes, Mogeeb A. Elsheikh designed a new type of wheel by studying the serpentine curve. Compared with other types of wheels, the curved outer edge type of wheel has good performance on sand [14]. Cai Qing designed a bionic grasping plate for seabed sediments and studied the stress–strain relationship and deformation characteristics of seabed soils, as well as the effects of different tool shapes on the rheological properties of soils, which provided guidance for the design of soil-touching components [15]. Using numerical simulation, Gang He investigated the effect of the cross-sectional parameters of a rigid footplate with holes and complete planes of the same cross-sectional area on the resistance during settlement. The study showed that annular footplates have a higher vertical load-bearing capacity than smooth foot plates, which provides a reference for the optimization of the foot of legged robots working in soft soil conditions [16].

In the field of agricultural machinery, Liang Tian established an interaction model between weeder wheels and paddy soil. Additionally, he conducted discrete element simulation and field experiments, and the results showed that the wheel could meet the requirements of a paddy field [17]. Zhewu Chen proposed a new type of curved-rim wheel suitable for paddy fields in which finite elements were used to simulate the interaction between the wheel and soil for wheel improvement design. The driving performance is analyzed, and satisfactory results are obtained [18]. Yongzhi Zhang designed a bionic blade of a paddy wheel based on the geometrical features of buffalo hooves. The experimental results showed that, compared with a normal blade, a bionic blade can improve the driving force of a wheel by resisting and reducing the impacts caused by sharp velocity variations in the water flow on discontinuous surfaces [19]. Gangdun Hu proposed an integral model for the interaction between a C-legged wheel and soil, which provided support for the establishment of a mathematical model for wheel–soil interactions [20]. Fumihiko Asano verified that the walking performance of a combined rimless walking robot can be improved by introducing an active oscillating mass to increase walking speed and stability [21]. Friedrich Pfeiffer explored the mechanical and control systems of a hexapod walking robot by employing biological principles and drew conclusions about design guidelines and drive system selection [22]. Bohumir Jelinek simulated vehicle motion and soil mechanical behavior in granular media using the discrete element method (DEM), providing a reference for improving vehicle design and soil mechanics models [23]. Kenji Hashimoto investigated the use of a WS-2 bipedal robot with wheeled feet to cope with bipedal walking and wheeled locomotion. It was experimentally verified that switching between bipedal walking and wheeled locomotion on different terrains can reduce the energy consumption of a robot [24].

In general, most of the studies on tractability improvement for soft ground focus on sandy ground, and relatively few studies have been conducted on paddy soil, which mainly changes the shape of the wheel based on biological or mechanical models. Therefore, in this paper, to improve the trafficability of wheels in soft and muddy soils such as paddy fields, a bionic walking wheel is designed based on the structural morphology and movement mode of the feet of the waders living in marshes and mudflats similar to those of the muddy soil of paddy fields. The bionic walking wheel adopts the arrangement of double-row wheel legs and staggered arrays to imitate the walking posture of waders. The two legs move alternately, cooperate with each other, and improve the smoothness of movement. The cam inside the bionic walking wheel is used to control the movement mode of the feet. The flippers open before touching the ground to increase the contact area and reduce sinking, and the toes bend and grip the ground while touching the ground to increase traction. Multi-rigid-body dynamics software (Adams View 2020) is used to simulate the movement of the wheel during the wading process, and the movement coordination and interference between the wheel legs are analyzed. The interaction between the bionic walking wheel and muddy soil was analyzed via coupled EDEM–ADAMS simulation, and the simulation analysis and experiments were conducted and compared with those for a common paddy wheel. By analyzing the effect of toe grip on traction, it was found that the soil under the feet can be disturbed to provide greater traction when the toe is bent downward. This study provides a reference for improving the trafficability of walking mechanisms in soft and muddy soils, such as paddy fields.

## 2. Bionic Design

### 2.1. Analysis of the Biological Prototype

**Figure 1:**
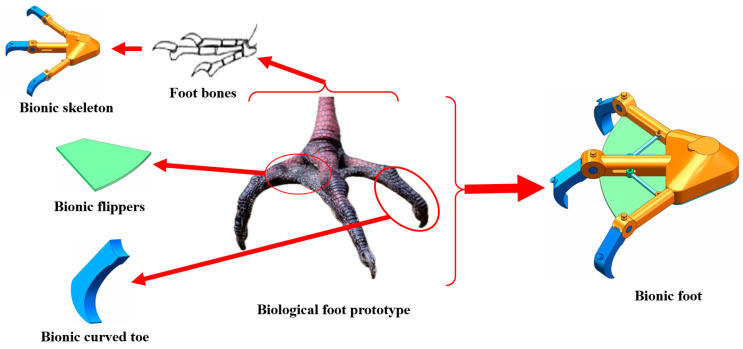
Bionic structural design of the toe of a walking wheel.

Waders are large birds that inhabit soft-ground environments such as wetlands, grasslands, and rice paddies. The legs of these waterfowl are usually relatively long and solid, making them especially suited to walking in shallow water without having to go deeper. The morphology of their feet and their form of locomotion are key to their ability to survive in soft and muddy soils. Wading birds also have relatively long toes, and their flippers have a semi-webbed structure [25]. This structure helps them maintain balance while walking in wetland environments and forage more efficiently. In addition, wading birds have a unique form of leg and foot movement that ensures high tractability over a variety of terrains. When they walk on soft ground, they begin by lifting one leg and pointing the toes backward. This action helps to reduce resistance on soft ground and prevents the toes from getting stuck in the mud. As the toes of the raised leg approach the ground, waders gently place their toes on the ground while opening their flippers to stretch their toes to increase the area of contact with the ground. Next, they will lightly touch the ground with the ends of their toes to help detect whether the ground is safe for continuing walking. When the decision is made to continue walking, weight is gradually transferred to that foot to maintain balance. This can be accomplished by squatting or slightly bending the ankle, which in turn spreads the weight. Moreover, the toes grip the ground during stride, providing better traction and preventing slipping. This makes it easier to move the body and reduces the risk of falling down due to instability [26,27]. The ability of the toes to grip the ground also increases their stability and helps them maintain balance, which is crucial for walking on soft ground or maintaining a standing position. Reverse engineering was used to model the structure of the biological foot, and this design focuses on the bionic study of the locomotion and structure of the foot in wading birds. Figure 1 shows the bionic structural design of the toe of a walking wheel.

### 2.2. Design of the Walking Wheel

Optimization of parameters is crucial in mechanical design [28,29]. The number of wheel legs largely determines the working performance of the walking wheel, and its reasonable selection requires comprehensive consideration of various factors. For walking wheels with the same diameter, reducing the number of wheel legs helps to simplify the structure, thus reducing the manufacturing costs. However, if the number of wheel legs is too small, this may lead to a decrease in the smoothness of the walking wheel. The starting resistance increases, which in turn requires additional starting power. Therefore, to ensure the necessary span between the wheel legs, the number of wheel legs should be reasonably selected according to the driving radius (i.e., the distance between the center of the wheel and the ground). Considering the previous research and design experience of walking wheels, it is more appropriate to control the number of wheel legs to be between 6 and 12 [30]. It can be adjusted according to the size of the driving radius and the specific requirements for wheel smoothness. In actual design, considering different working environments and task requirements, the number of wheel legs can be flexibly adjusted according to the specific situation.

In this paper, the driving radius of the walking wheel is 320 mm, and the hub radius is 125 mm. The number of single-row wheel legs is reasonably chosen to be 6 when considering the size of the driving radius. To further improve the smoothness of movement, a walking wheel with double-row wheel legs is designed based on the bipedal structure of the bionic prototype. The staggered angle between the two rows of walking wheels is set at half of the angle between the two legs of the single row. This design is inspired by the walking posture of waders, which enables two rows of wheel legs to move in concert and alternate, thus effectively improving the smoothness of the overall movement.

**Figure 2:**
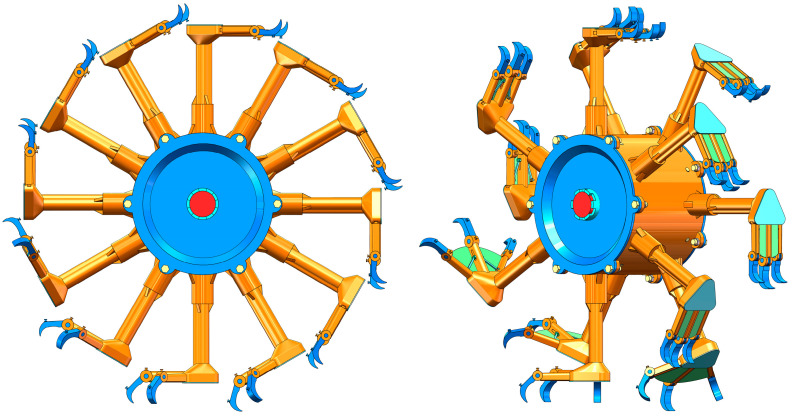
Overall structure of the bionic walking wheel.

The design of the wheel foot is inspired by the movements of the waterfowl. Before the foot touches the ground, the wheel foot first spreads the toes while opening the flippers to increase the contact area. When the toes play a primary supporting role, they flex and grip the ground to increase overall stability and traction. Figure 2 illustrates the overall structure of the bionic walking wheel.

**Figure 3:**
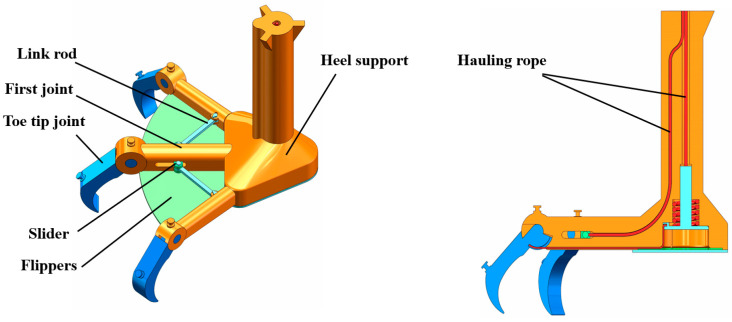
Structure of the wheel foot.

As a key component of the walking wheel, the size, morphology, and movement mode of the foot directly determine the performance of the walking wheel. Inspired by the foot morphology and movement mode of wading birds, we designed a bionic foot to slow sinking and increase the traction force. Figure 3 shows the structure of the bionic walking wheel’s foot. The overall shape is presented as a three-toe structure consisting of the toes and heel support. The foot has two states: the touchdown state and the off-ground state. When the walking wheel is off the ground, the flippers are closed, and the toes are straight, providing no traction. When touching the ground, the flippers are open, and the toes are flexed, allowing the toe joints to stab into the soil, achieving the goal of increasing traction and reducing sinkage.

**Figure 4:**
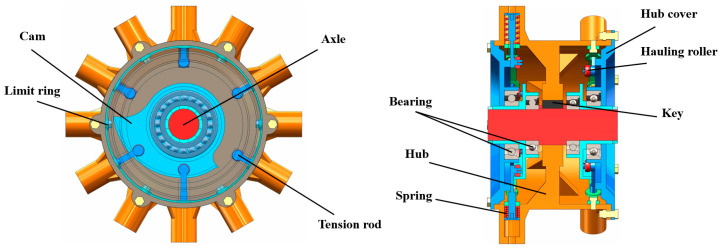
Structure of the wheel hub.

The wheel hub mainly supports and transmits power. The center bearing plate of the wheel hub is connected to the wheel axle through the key connection to transfer power. The rear end of the wheel leg and the shell are integrated structures to increase stability, and the front and rear end connections of the wheel legs are equipped with reinforcements to increase the pressure-bearing capacity. Figure 4 shows the structure of the bionic walking wheel’s hub.

### 2.3. Working Principle of the Walking Wheel

**Figure 5:**
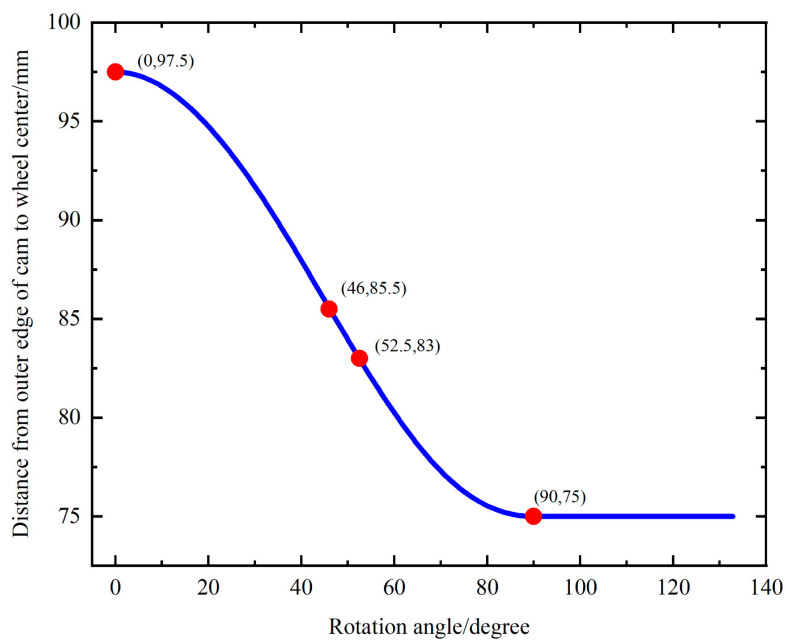
Distance from the outer edge of the cam to the center of the walking wheel.

When the walking wheel moves forward, a single wheel leg rotates one circle around the center of the walking wheel. The walking wheel can be divided into three gaits according to the working state. Figure 5 illustrates the distance from the outer edge of the cam to the center of the walking wheel. 0° indicates that the rotation starts from the left end of the cam. From 0° to 46°, the distance between the outer edge and the wheel center changes from 97.5 mm to 85.5 mm. Through the small roller at the front of the tension rod, the tension rod can be stretched upward by 12 mm. The spring between the tension rod and the limit ring is compressed at this time. The tension rod pulls the hauling rope that controls the opening and closing of the flippers upward by the same length. The flippers are controlled to open and hold before touching the ground, while the tension band at the front of the first joint of the foot is stretched and deformed to create a certain amount of tension. The opened flippers increase the contact area with the ground to reduce sinkage. This is the first gait cycle.

The second gait pattern occurs from 46° to 90°. The spring between the tension rod and the limit ring is compressed once more. It is the empty stroke from 46° to 52.5° where the hauling rope does not produce downward tension, and the state of the toe joint does not change. From 52.5° to 90°, the tension rod rises 8 mm and pulls the hauling rope that controls the bending of the toe joint by the same distance. It controls toe joint bending by pulling the elastic band between the toe joint and the first joint to produce a certain tension. Therefore, the toe joints and footplate exert pressure on the soil, and when the toe bends to the maximum pressure, the wheel leg is perpendicular to the ground, while the soil under the foot is subjected to the greatest downward pressure. Together with the force exerted on the soil by toe bending, these forces enhance the temporary immobilization of the soil and the traction of the walking wheel. This is the second gait. This phase is more gentle because the toe contacts the soil, creating some resistance to reduce impact.

From 90° to 135°, the distance between the outer edge of the cam and the center of the walking wheel remains constant, and the gripping action continues. From 135° to the next cycle, the outer edge of the cam disappears. Under the action of the spring force between the limit ring and the tension rod, the tension rod returns to its original position, and the hauling rope relaxes. The foot is returned to its original state by means of an elastic band to prepare it for the next cycle of movement. This is the third gait.

## 3. Modeling of Wheel–Soil Interactions

### 3.1. Modeling of Wheel–Soil Interactions

The interaction between the walking wheel and the paddy soil is a key and important factor in the performance of this design. This design mainly analyzes the interaction between the bionic walking wheel and the paddy soil and compares it with the traction force suffered by the common paddy wheel. To ensure that the simulation data are consistent with the actual situation, a contact model was determined prior to discrete element (DEM) simulation analysis [31]. By reviewing the information about the interaction between paddy soil and walking mechanisms [32], various parameters of the simulation system were determined, and a three-dimensional EDEM model of paddy soil was established.

The Hertz–Mindlin [33] and Johnson–Kendall–Roberts (JKR) cohesion models [34] in the software EDEM (EDEM 2021) used in this paper are cohesion models that can take into account the effect of van der Waals forces in the contact region. This approach can simulate the mechanical interactions between water-containing particles well and is suitable for simulating paddy soils. In the simulation process, the normal force, tangential force, normal damping, tangential damping, and rolling friction between particles and between particles and between particles and the wheel are considered. In the Hertz–Mindlin model with JKR, the tangential component is based on the theory of Mindlin–Deresiewicz, the normal component is based on the theory of Johnson, Kendal, and Roberts with Hertz, and the rolling friction is modeled as a contact-independent directional constant torque [35,36]. It is assumed that the deformation of the particles is elastic and that the normal interaction is not affected by the tangential interaction [37,38]. The modeled forces in EDEM are calculated as shown below.

In the JKR contact model, the JKR normal force is calculated using Equations (1) and (2).

(1) $$F_{JKR}=-4\sqrt{\pi \gamma E^{*}}\alpha^{\frac{3}{2}}+\frac{4E^{*}}{2R^{*}}\alpha^{3}$$

(2) $$\delta =\frac{\alpha^{2}}{R^{*}}-4\sqrt{\frac{4\pi \gamma \alpha }{E^{*}}}$$

where δ is the overlap, γ is the surface energy, $E^{*}$ is the equivalent Young’s modulus, $R^{*}$ is the equivalent radius, and α is the ratio of the contact radius to the equivalent radius. Parameters are calculated using Equations (3) and (4).

(3) $$\frac{1}{E^{*}}=\frac{\left(1-v_{i}^{2}\right)}{E_{i}}+\frac{\left(1-v_{j}^{2}\right)}{E_{j}}$$

(4) $$\frac{1}{R^{*}}=\frac{1}{R_{i}}+\frac{1}{R_{j}}$$

where $E_{i}$, $v_{i}$, $R_{i}$, $E_{j}$, $v_{j}$, and $R_{j}$ are Young’s modulus, Poisson’s ratio, and the radius of each contact sphere, respectively.

When γ=0, the surface energy disappears, and the Hertz–Mindlin normal force is reached, as calculated by Equation (5).

(5) $$F_{Hertz}=\frac{4}{3}E^{*}\sqrt{R^{*}}\delta^{\frac{3}{2}}$$

The total normal force on the particles $F_{n}$ is calculated by Equation (6).

(6) $$F_{n}=F_{Hertz-Mindlin}+F_{JKR}$$

The Mindlin tangential force is calculated by Equation (7).

(7) $$F_{t}=-S_{t}\delta_{t}$$

where $\delta_{t}$ is the tangential overlap and $S_{t}$ is the tangential stiffness, calculated by Equation (8).

(8) $$S_{t}=8G^{*}\sqrt{R^{*}\delta_{n}}$$

where $\delta_{n}$ is the normal overlap and $G^{*}$ is the equivalent shear modulus, calculated by Equation (9).

(9) $$G^{*}=\frac{1}{2}{\left(\frac{1}{G_{i}}+\frac{1}{G_{j}}\right)}^{-1}$$

The normal damping force $F_{n}^{d}$ is calculated by Equation (10).

(10) $$F_{n}^{d}=-2\sqrt{\frac{5}{6}}\beta \sqrt{S_{n}m^{*}}v_{n}^{\bar{rel}}$$

where $m^{*}$ is the equivalent mass, $s_{n}$ is the normal stiffness, $v_{n}^{rel}$ is the normal component of the relative velocity, and β is the damping ratio, calculated by Equations (11)–(13).

(11) $$m^{*}={\left(\frac{1}{m_{1}}+\frac{1}{m_{i}}\right)}^{-1}$$

(12) $$S_{n}=2E^{*}\sqrt{R^{*}\delta_{n}}$$

(13) $$\beta =\frac{-\ln e}{\sqrt{\ln^{2}e+\pi^{2}}}$$

where e is the coefficient of restitution.

The tangential damping force $F_{t}^{d}$ is calculated by Equation (14).

(14) $$F_{t}^{d}=-2\sqrt{\frac{5}{6}}\beta \sqrt{S_{t}m^{*}}v_{t}^{\bar{rel}}$$

where $v_{t}^{rel}$ is the tangential component of the relative velocity.

It is also important to simulate rolling friction, which is handled by applying a contact-independent torque with a constant direction to the contact surface, as shown in Equation (15).

(15) $$\tau_{t}=\pm \mu_{r}F_{n}R_{d}$$

where $\mu_{r}$ is the coefficient of rolling friction and $R_{d}$ is the distance from the contact point to the center of mass. When the angular velocity of the object at the contact point is counterclockwise, the formula takes a negative sign, and when it is clockwise, the formula takes a positive sign. In addition, the JKR model provides attractive cohesion; even if the particles are not in direct contact, they have a certain attractive force. They can be calculated by Equations (16) and (17).

(16) $$\delta_{c}=-\sqrt{4\pi \gamma \alpha_{c}/E^{*}}+\frac{{\alpha_{c}}^{2}}{R^{*}}$$

(17) $$\alpha_{c}={\left[\frac{9\pi R^{*2}}{2E^{*}}\left(\frac{3}{4}-\frac{1}{\sqrt{2}}\right)\right]}^{\frac{1}{3}}$$

where $\delta_{c}$ is the normal overlap caused by the attractive force and $\alpha_{c}$ is the effective radius when there is an attractive force.

The soil simulation parameters in EDEM are shown in Table 1. Since the paddy soil is clayey and its particle distribution is more regular, it is assumed that the particles of the paddy soil are spherical and the sizes are the same. To make the calculation convenient and ensure that the error is within a reasonable range, the radius of the particles is selected as 8 mm. The density, shear modulus, and Poisson’s ratio are inherent properties of the soil and are determined by checking the parameters of the paddy soil in northern China. The surface energy of paddy soil varies greatly from region to region, and this value is taken as the surface energy of paddy soil in the experimental field of Shenyang Agricultural University. There is a strong relationship between the time step size and the calculation accuracy in EDEM, so an appropriate time step size is selected to save time.

The interaction coefficients between the soil and wheels are shown in Table 2. Due to the viscous effect of soil particles in paddy fields, when a wheel collides with soil or when the soil collides with the soil, a small restitution coefficient occurs under viscous conditions, which has a small impact on the simulation, and the restitution coefficient is estimated to be 0.05 by consulting the literature. Sliding friction is the main form of friction between the wheel and soil. Taking into account the viscosity of the soil and the material of the wheel, the sliding friction coefficient is selected to be 0.5. Because of the cohesion between the soil particles, sliding between the soil particles is difficult, resulting in a large coefficient of sliding friction; the sliding friction coefficient between the soil particles is selected to be 0.9. Because paddy soil is a wet soil, a water film will form between particles, which leads to a small coefficient of rolling friction between the soil particles and wheels and between the soil particles and soil particles; this coefficient is taken as 0.01.

### 3.2. Dynamics Analysis of the Walking Wheel

The double-row bionic walking wheel designed in this paper is mainly applied to soft ground. The movement of the foot is controlled by the cam, and the movement of the parts inside the wheel is regular. The shape and size of each component were adjusted to avoid interference. Considering the double-row structure, an interference check is required to verify that the spacing between the two rows, as well as the length of the toes, are appropriate for preventing collisions during movement. During the movement of the walking wheels, there is a possibility of interference between the nearest two feet. Due to its own law of motion, it is only necessary to verify that the nearest pair of toes do not interfere with each other during movement.

**Figure 6:**
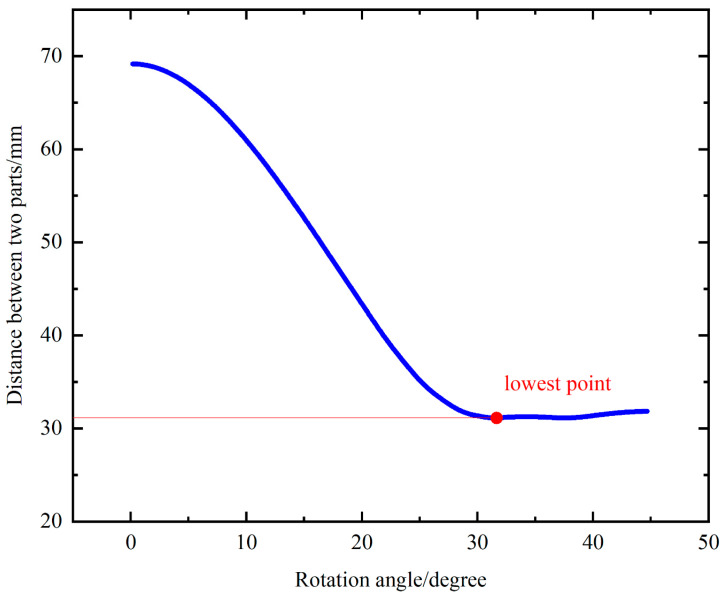
Distance between two parts during movement.

Multi-rigid-body dynamics analysis software (ADAMS) (Adams View 2020) was used to simulate the movement of the toes during the wading process. The coordination of motion and interference of the wheel are analyzed. Figure 6 shows the distance between the two parts during movement. The figure shows that the minimum distance between two parts is greater than 0 when the flippers are spread out to their maximum and the toes are not flexed; therefore, no interference occurs.

**Figure 7:**
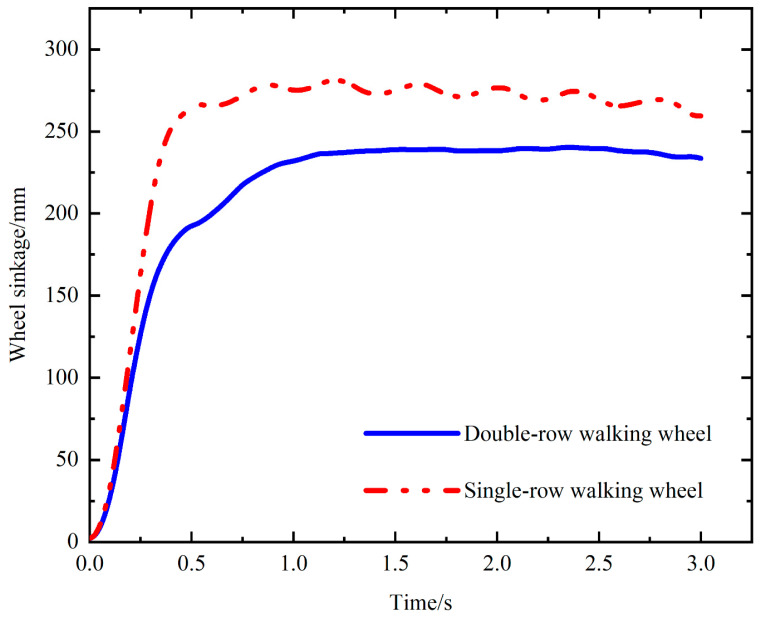
Comparison of the smoothness and sinkage during the movement of single-row and double-row walking wheels.

The motion smoothness of the walking wheel is a critical concern due to potential bumps, which can adversely affect the overall machinery, including loose screws and damaged pars. This paper employs an EDEM–ADAMS coupling simulation to simulate the motion state of a walking wheel in a real situation, and a comparative analysis of smoothness is carried out. Figure 7 shows the comparison of smoothness and sinkage during the movement of a single-row walking wheel and a double-row walking wheel.

As shown in Figure 7, 0–0.5 s indicates the process in which the walking wheel is subjected to gravity and gravity-directed loads, descends from the soil surface to the hard bottom, and achieves stable motion. During this phase, the walking wheel exhibits a significant displacement in its center of gravity. At 0.5–1.25 s, the movement of the single-row and double-row walking wheels tends to stabilize. Subsequently, at 1.25–3 s, the displacement of the center of gravity of the double-row walking wheel is nearly linear. This stability can be attributed to the double-row structure of the wheel, which enhances its continuity compared to that of single-row wheels; thus, the double-row walking wheel shows good smoothness. In contrast, for a single-row structure, the displacement of the center of gravity fluctuates, resulting in bumps. This phenomenon can be ascribed to the poor continuity of the outer edge of the single-row structure compared to that of the double-row structure. In addition, in terms of sinkage, the double-row structure has shallower sinkage than the single-row structure due to the larger contact area of the double-row wheel foot. Overall, the double-row structure outperforms the single-row structure in terms of smoothness and sinkage.

## 4. Traction Trafficability Analysis

### 4.1. EDEM–ADAMS Coupling Simulation Analysis

The interaction between the walking wheel and the muddy soil is analyzed by an EDEM–ADAMS coupling simulation, and comparative simulation analysis and experiments are conducted with a common paddy wheel to verify that the bionic walking wheel has superior traction and anti-sinking performance. By analyzing the effect of toe grip on traction performance, it was found that when the toe is bent downward, the soil under the foot can be disturbed to provide greater traction. The design model is imported into ADAMS, appropriate constraints and drivers are added to perform the bidirectional coupling simulation of ADAMS-EDEM, and a soil trough is modeled to simulate the interaction between the walking wheel and the soil. This approach aims to more accurately simulate the performance of a walking wheel in practice and provide a reference for design optimization.

**Figure 8:**
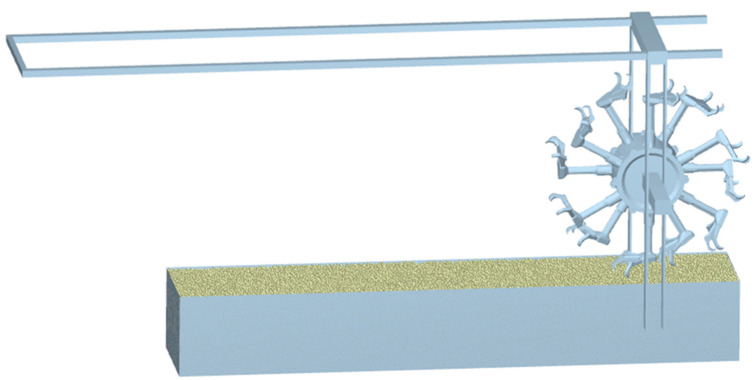
Soil trough simulation.

In this paper, a load in the direction of gravity is applied to the center of gravity of the walking wheel so that the wheel is subjected to a total force of 350 N along the direction of gravity to simulate the actual situation, and the speed of the wheel is 25 r/min. To more clearly highlight the tractability of the bionic walking wheel in paddy fields, we deploy a comparative analysis with a common paddy wheel with the same driving radius. The simulation results of the bionic walking wheel are compared with those of commonly used paddy wheels. Figure 8 shows the soil trough simulation.

**Figure 9:**
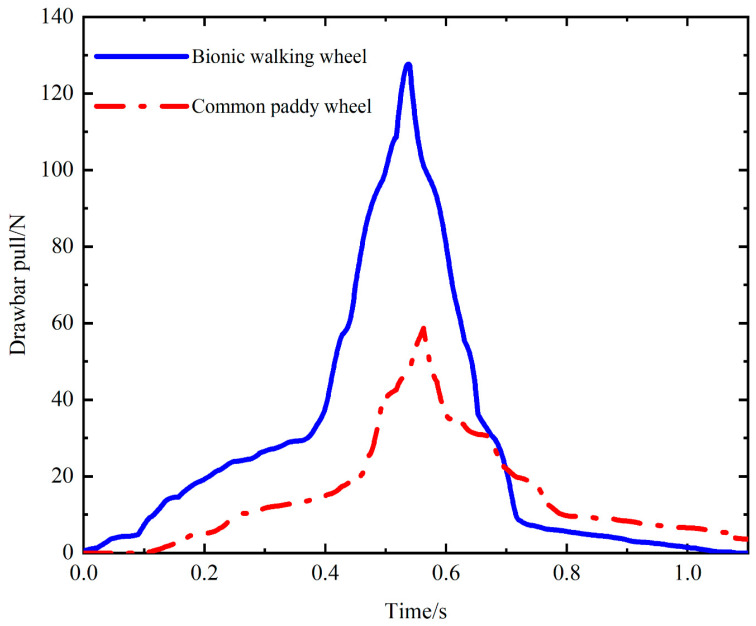
Drawbar pull of the bionic walking wheel compared to that of the common paddy wheel.

Figure 9 shows the comparison between the drawbar pull of the bionic walking wheel and the common paddy wheel with the same dynamic parameters. It is clear that the drawbar pull exerted on the bionic walking wheel is greater than that exerted on the common paddy wheel, which indicates that the bionic walking wheel has superior traction performance in paddy soil compared to the common paddy wheel under the same circumstances. In addition, an increase in the slope of the drawbar pull curve is observed at 0.4–0.6 s, which is due to the irregular outer edges of both wheels, which cause most of the gravitational force to suddenly act on this wheel foot when replacing the main supporting wheel foot, making the soil reaction force on this wheel foot instantly larger.

**Figure 10:**
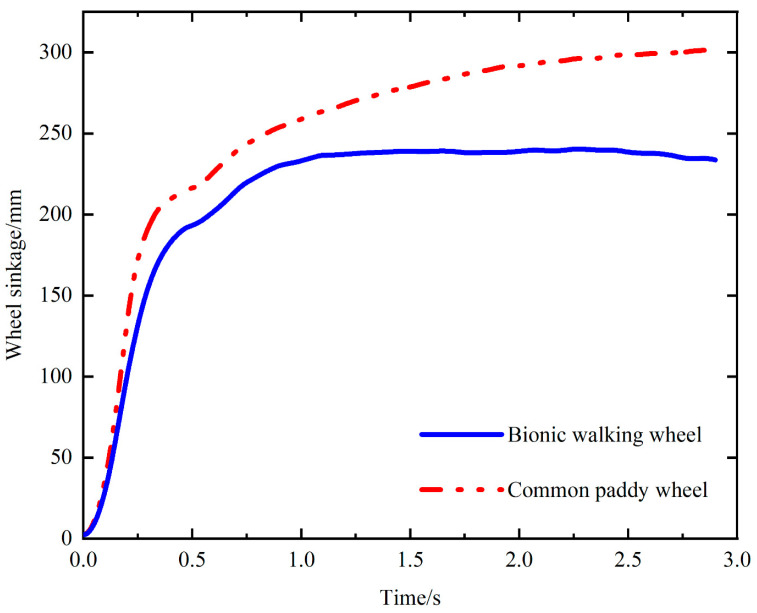
Sinkage of the bionic walking wheel compared to that of the common paddy wheel.

Figure 10 shows a comparison between the sinkage of the bionic walking wheel and that of the common paddy wheel. The displacements of the bionic walking wheel and the paddy field wheel in the direction of gravity are compared. The results showed that the sinkage of the common paddy wheel was much greater than that of the bionic walking wheel. Since deeper sinkage increases the resistance to moving forward, a common paddy wheel is not suitable for large loads. In contrast, the bionic walking wheel is able to withstand greater loads under normal working conditions, highlighting its advantages in traction and anti-sinking performance.

### 4.2. Effect of Toe Gripping on Traction Performance

In this paper, a comparative simulation with and without toe gripping is designed to investigate the effect of toe gripping on traction performance. The two sets of simulations remain consistent in terms of parameters such as load and speed, with the only difference being the motion state of the toes. Such a comparative analysis will help to gain insight into the role of toe gripping in the traction performance of bionic walking wheels.

In the first set of experiments, the toes began to flex before the leg was perpendicular to the ground and continued to reach a maximum degree of flexion until the leg was perpendicular to the ground. This simulates the action of a bionic walking wheel when gripping the ground. We will observe how the traction changes in this case. In the second set of experiments, the toes were set to remain upright without flexion. This allows us to compare the differences in performance between toe-gripping and non-gripping motions, thereby providing deeper insights into the effect of toe-gripping on the traction performance of bionic walking wheels.

**Figure 11:**
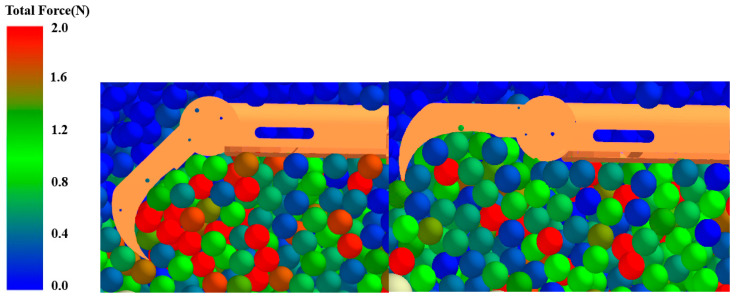
Comparison of forces on soil particles with and without toe flexion.

**Figure 12:**
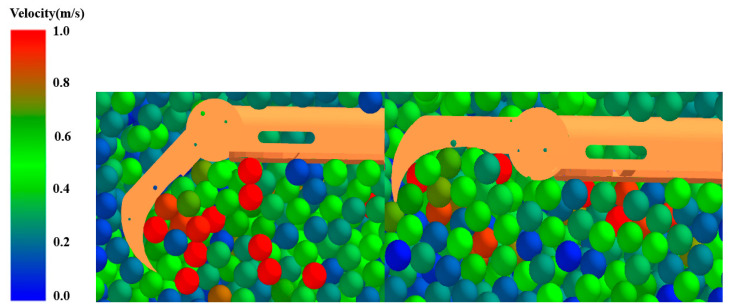
Comparison of the velocities of soil particles with and without toe flexion.

The soil particles affected by the walking wheel are analyzed via EDEM sectioning, taking the symmetry plane of the foot as the section. The effect of the middle toe on the soil particles can be visualized, with the other toes having nearly the same effect as the middle toe. Figure 11 and Figure 12 show a comparison of the effect of whether the toe is flexed on the force and velocity of the soil particles, with the color of the particles representing the size of the force. When the toe bends, the particles that are subjected to greater forces are denser in the concave region formed by flexion. This is because the bending of the toe squeezes the soil particles toward the heel direction and, together with the pressure applied to the soil by the plantar, produces a temporary immobilization effect on the soil under the foot, which provides greater traction. When the toe does not bend, the distribution of soil particles with a large force is looser, and the effect of squeezing the soil is not obvious compared to that of the bending toe. The traction provided to the walking wheel was lower than that provided when the toes were bent, indicating that greater traction can be provided when the toes are bent. In terms of the velocity of the particles, there were more particles with a certain velocity near the bent toe than near the unbent toe, indicating that the bent toe can increase the amount of soil disturbance and make it subjected to a greater force given by the soil.

**Figure 13:**
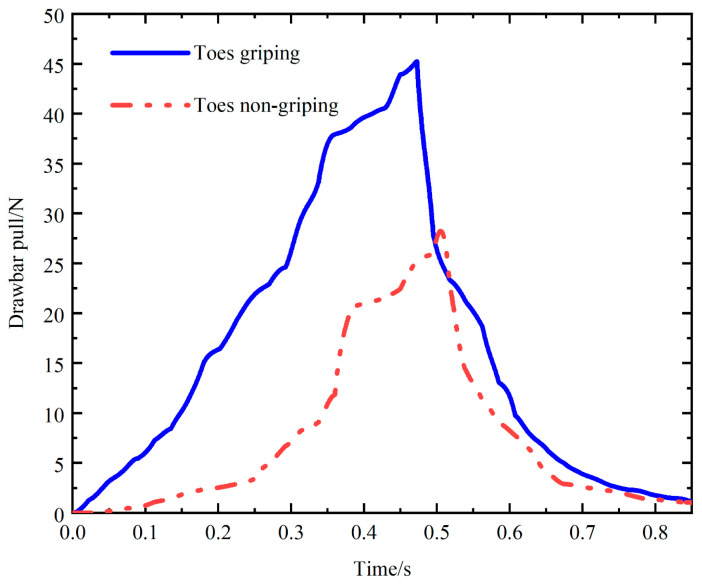
Effect of whether the toes are flexed or not on drawbar pull.

Figure 13 demonstrates the effect of whether the toe is flexed on traction performance. The slopes of the two curves are smaller at 0–0.3 s, and they increase significantly from 0.3 s to 0.5 s due to the change in the supporting leg. The overall drawbar pull curve for toe gripping was greater than that for toe nongripping. This finding showed that the bent toe could provide greater drawbar pull during movement. This is because when the toe is bent, the soil under the foot has strong temporary immobilization performance, and the soil will provide more reaction force.

### 4.3. Wheel–Soil Interaction Experiments

**Figure 14:**
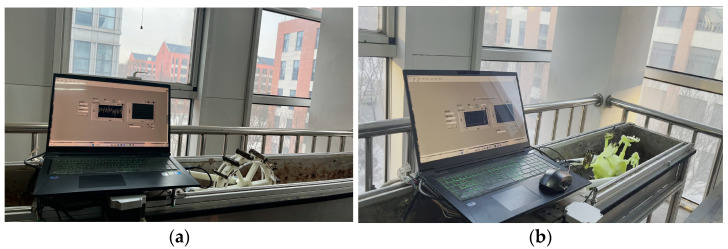
Wheel–soil interaction experiments: (**a**) common paddy wheel and (**b**) bionic walking wheel.

To verify the traction trafficability of the bionic walking wheel in the soft and muddy soils of paddy fields, experiments were conducted based on the soil trough testing system and were compared with those of common paddy wheels, as shown in Figure 14. The test soil was obtained from the soil in the paddy field of Shenyang Agricultural University. The surface of the soil trough should be as flat as possible and thick enough before the experiment starts. The bionic walking wheel and the common paddy wheel were connected to the platform in stages, and their initial positions were adjusted. The drawbar pull was measured by a six-dimensional force/torque sensor, i.e., the horizontal component of the wheel’s forward direction. The sinkage was measured by a linear displacement sensor. The total weight of the wheel and its load are measured by a six-dimensional force/torque sensor and adjusted by adding or subtracting weights.

**Figure 15:**
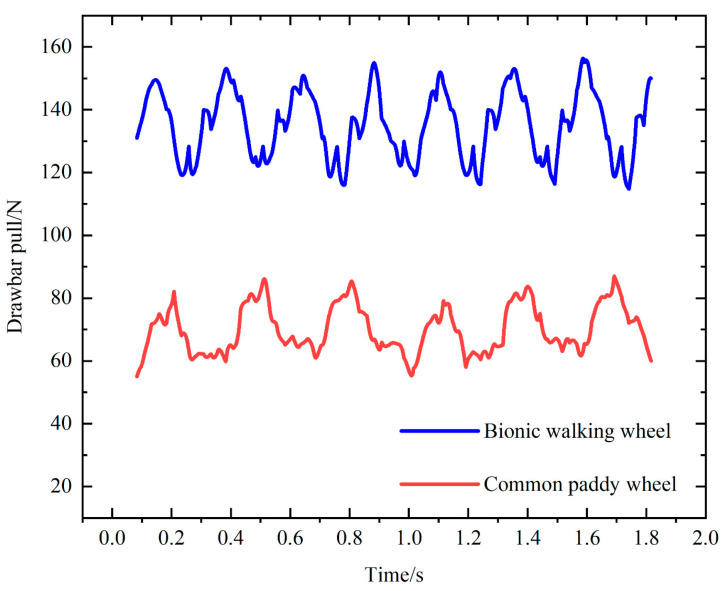
Comparison of drawbar pull between the bionic walking wheel and common paddy wheel.

Figure 15 shows the comparison of the drawbar pull between the bionic walking wheel and the common paddy wheel during movement. The drawbar pull of the bionic walking wheel is greater than that of the common paddy wheel, and the maximum drawbar pull of the bionic walking wheel is 113.56% greater than that of the common paddy wheel. This indicates that, under the same ground conditions, the bionic walking wheel has better traction trafficability. In addition, as shown in Figure 15, there are fluctuations in the drawbar pull because the wheel legs/lugs are spaced apart. There are periodic variations in the force on the soil under the wheel during rolling, and the drawbar pull on the wheels is thus subjected to periodic variations, indicating that the distribution density of the wheel legs/lugs has an effect on the traction performance.

**Figure 16:**
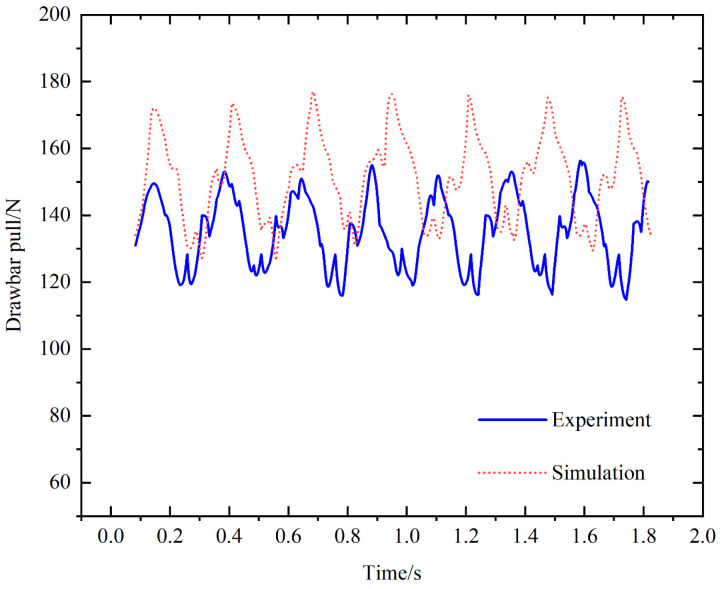
Comparison of drawbar pull between experimental data and simulation data.

In addition, to verify the accuracy of the simulation model of the bionic walking wheel-paddy soil interaction established in this paper, the experimental data of drawbar pull were compared with the simulation data, as shown in Figure 16. The results show that the simulation data are in good agreement with the experimental data, which verifies the correctness of the model.

**Figure 17:**
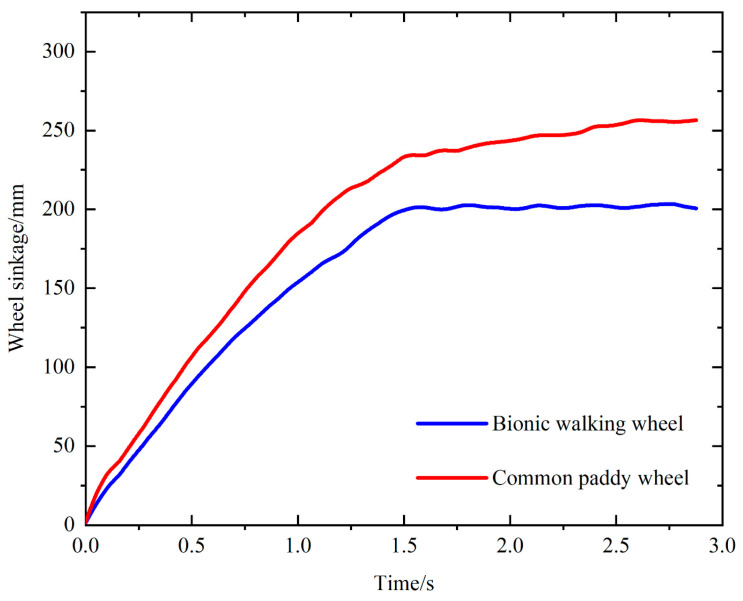
Comparison of sinkage between the bionic walking wheel and the common paddy wheel.

The experimental results of the wheel sinkage are shown in Figure 17. In the beginning stage, both the bionic walking wheel and the common paddy wheel sink faster, because they begin to sink from the soil surface to the hard bottom. The soil gives less support in the beginning stage, which leads to faster sinking speed. Then, the sinkage tends to stabilize. It can be clearly seen that the sinkage of the common paddy wheel is larger than that of the bionic walking wheel. Therefore, it indicates that the bionic walking wheel has a better anti-sinking performance compared with that of the common paddy wheel.

**Figure 18:**
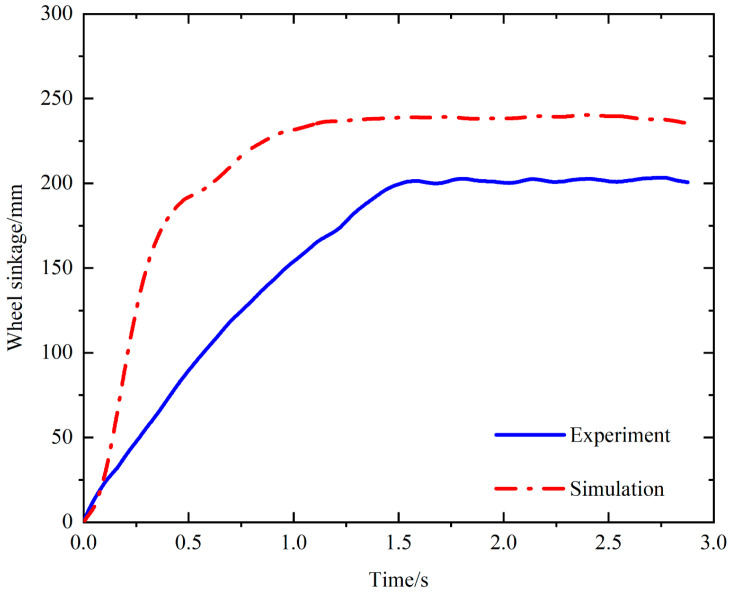
Comparison of sinkage between experimental data and simulation data.

Figure 18 shows the comparison between the experimental data and the simulation data of the sinkage of the bionic walking wheel. It can be observed from the curve that their overall trends are basically the same. The difference is that the sinking speed of the simulated bionic walking wheel is faster at the beginning. This is because the simulated soil particles are much larger than the real soil particles, so the pores of the simulated soil are larger. This leads to a large difference in the initial stage of the sinking speed, and also leads to the final sinkage of the simulation being greater than that of the experiment.

## 5. Conclusions

In view of the complex and special physical and mechanical properties of paddy soil, conventional wheels have the disadvantages of severe slippage, sinking, and low traction performance. To improve the wheels’ trafficability in soft and muddy soils, a bionic walking wheel is designed based on the structural morphology and movement mode of the feet of the waders living in marshes and mudflats, similar to the muddy soil of paddy fields. The bionic walking wheel adopts the arrangement of double-row wheel legs and staggered arrays to imitate the walking posture of waders. The two legs move alternately, cooperate with each other, and improve the smoothness of movement. The flippers open before touching the ground to increase the contact area and reduce sinking, and the toes bend and grip the ground while touching the ground to increase traction. ADAMS is used to simulate the movement of a wheel during the wading process, and the movement coordination and interference between the wheel legs are analyzed. The simulation results show that there is no interference between the parts and that the movement smoothness is good. The interaction between the bionic walking wheel and muddy soil was analyzed via coupled EDEM–ADAMS simulation, and the simulation analysis and experiments were conducted and compared with those for a common paddy wheel. The results showed that the bionic walking wheel designed in this paper improved the drawbar pull by 113.56% compared with that of a common paddy wheel and had better anti-sinking performance. By analyzing the effect of toe grip on traction, it was found that the soil under the feet can be disturbed to provide greater traction when the toe is bent downward. This study provides a reference for improving the trafficability of walking mechanisms in soft and muddy soils, such as paddy fields. In the future, we will further optimize the wheel structure to increase the adaptability to the rough and muddy soil environment in paddy fields, taking into account crop/vegetation disturbances and obstacles to improve its trafficability and adaptability.

## Figures and Tables

**Table 1:** Soil modeling parameters in EDEM.

Parameters	Values
Soil particle radius (mm)	8
Soil density (kg/m^3^)	2400
Soil shear modulus (Pa)	1 × 10^7^
Poisson’s ratio	0.25
Surface energy (J/m^2^)	0.15
Time step size (s)	6 × 10^−5^

**Table 2:** Coefficients of soil–wheel interaction.

	Coefficient of Restitution	Coefficient of Sliding Friction	Coefficient of Rolling Friction
Wheel–Soil	0.05	0.5	0.01
Soil–Soil	0.05	0.9	0.01

## Data Availability

Data are contained within the article.
